# Automatic method for individual parcellation of manganese-enhanced magnetic resonance imaging of rat brain

**DOI:** 10.3389/fnins.2022.954237

**Published:** 2022-07-28

**Authors:** Zhiguo Bao, Tianhao Zhang, Tingting Pan, Wei Zhang, Shilun Zhao, Hua Liu, Binbin Nie

**Affiliations:** ^1^First Affiliated Hospital of Henan University, Kaifeng, China; ^2^Beijing Engineering Research Center of Radiographic Techniques and Equipment, Institute of High Energy Physics, Chinese Academy of Sciences, Beijing, China; ^3^School of Nuclear Science and Technology, University of Chinese Academy of Sciences, Beijing, China; ^4^Physical Science and Technology College, Zhengzhou University, Zhengzhou, China

**Keywords:** individual parcellations, ROI-based analysis, manganese-enhanced magnetic resonance imaging (MEMRI), rat brain, stereotaxic template set

## Abstract

**Aims:**

To construct an automatic method for individual parcellation of manganese-enhanced magnetic resonance imaging (MEMRI) of rat brain with high accuracy, which could preserve the inherent voxel intensity and Regions of interest (ROI) morphological characteristics simultaneously.

**Methods and results:**

The transformation relationship from standardized space to individual space was obtained by firstly normalizing individual image to the Paxinos space and then inversely transformed. On the other hand, all the regions defined in the atlas image were separated and resaved as binary mask images. Then, transforming the mask images into individual space *via* the inverse transformations and reslicing using the 4th B-spline interpolation algorithm. The boundary of these transformed regions was further refined by image erosion and expansion operator, and finally combined together to generate the individual parcellations. Moreover, two groups of MEMRI images were used for evaluation. We found that the individual parcellations were satisfied, and the inherent image intensity was preserved. The statistical significance of case-control comparisons was further optimized.

**Conclusions:**

We have constructed a new automatic method for individual parcellation of rat brain MEMRI images, which could preserve the inherent voxel intensity and further be beneficial in case-control statistical analyses. This method could also be extended to other imaging modalities, even other experiments species. It would facilitate the accuracy and significance of ROI-based imaging analyses.

## Introduction

Numerous studies have demonstrated the usefulness of magnetic resonance imaging (MRI) techniques in animal experiments, which is important for pathogenesis researches, drug developments, and so on (Bible et al., 2012; Kim et al., 2012; Wang et al., 2012, 2018; Li et al., 2015; Liang et al., 2020; Tu et al., 2020). Manganese-enhanced MRI (MEMRI), a new MRI technique, could detect the active neurons and trace neuronal pathway by monitoring manganese ions (Mn^2+^) *in vivo* (Aoki et al., 2004; Koretsky and Silva, 2004). By tracing the deposition of Mn^2+^, the active neurons would have high voxel intensity in MEMRI images, while other voxels have low intensity. Recently, the MEMRI technique has been widely used in various studies of the rodent brain (Ho et al., 2018; Perez et al., 2018; Gimenes et al., 2019; Yang et al., 2020; Bearer et al., 2022).

Imaging analysis is a prerequisite in MEMRI studies for quantitative interpretations of neuronal dysfunctions. Regions of interests (ROIs)-based analysis is one of the most frequently used methods in rat brain MEMRI studies (Spurny et al., 2019). Similar to other modalities of MRI, by spatially normalizing individual MEMRI images into a standard space, ROIs could be extracted *via* a corresponding atlas image automatically (Nie et al., 2013; Barriere et al., 2019). However, it has been found in functional studies that the brain sub-regions vary across individuals (Chong et al., 2017; Salehi et al., 2020; Reijonen et al., 2021). Moreover, because of image interpolations and transformations in spatial normalization, the inherent MEMRI intensity would be confused by adjacent voxels inevitably (Ashburner and Friston, 1999; Zhilkin and Alexander, 2004). Especially, the voxel intensity of active neurons would be affected, even decreased, by adjacent un-activated neurons, and the statistical significance of case-control studies would further be affected (Lv et al., 2021).

Alternatively, manually tracing ROIs in individual images is another important method in MEMRI studies, which could avoid the influences of image normalization and preserve the inherent voxel intensity (Jackson et al., 2011). However, this method has many disadvantages, such as low accuracy, labor-intensive, subjective and poor robustness, and so on. Therefore, it would be highly desirable to develop an automatic method for ROI parcellations in individual space of MEMRI images of rat brain.

In addition, more than a decade ago, our lab had constructed an automatic atlas-based method for tracing ROIs in individual space by registering the template images to individuals (Nie et al., 2010). However, as the resolution of individual images was often lower than the template, the accuracy of this image registration was always lower than spatial normalization, so that the subtle ROIs were hard to trace out precisely.

Therefore, the current study was aimed to introduce a new automatic method for individual parcellation of MEMRI images of rat brain with high accuracy, and both the inherent voxel intensity and ROI morphological characteristics could be preserved simultaneously. For evaluation, individual parcellations were performed on two groups of T2-weighted (T2WI) and MEMRI images, and further comparing with the parcellations in standardized space. Moreover, the method constructed in this study was packaged and available by contacting the corresponding author at niebb@ihep.ac.cn.

## Materials and methods

### The individual parcellation method

**Figure 1:**
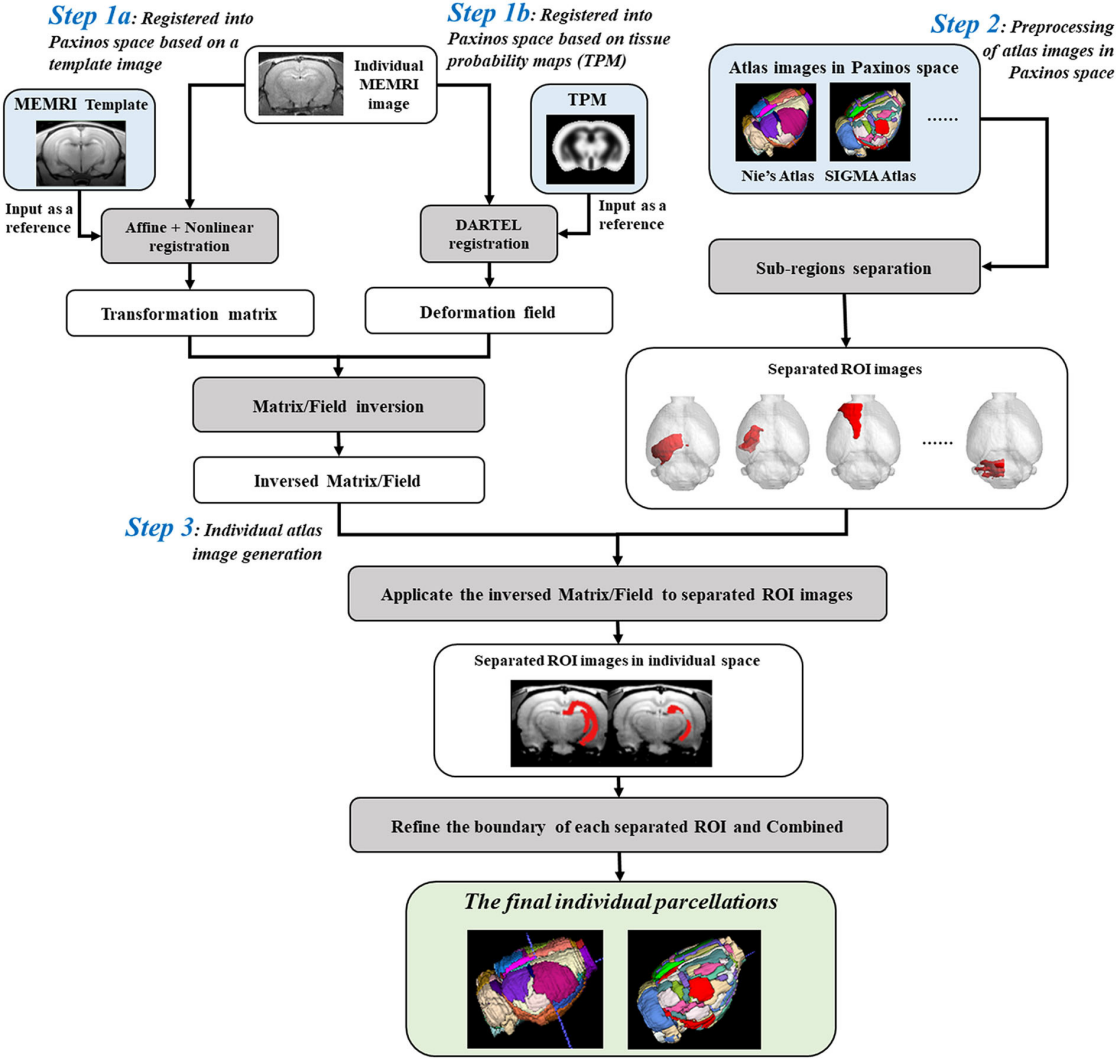
Flow chart for creating individual parcellations of manganese-enhanced magnetic resonance imaging (MEMRI) image of rat brain. *Step 1*: The individual MEMRI image was registered into Paxinos space either by affine/nonlinear transformations based on the MEMRI template image (*Step 1a*), or by DARTEL algorithm based on the tissue probability maps (TPM) (*Step 1b*), and the transformation matrix/deformation field was obtained, named as *Matrix*_*sn*_/*Deform*_*y*_. Inverse transformation of the *Matrix*_*sn*_/*Deform*_*y*_ was then calculated, named *InvMatrix*_*sn*_/*InvDeform*_*y*_. *Step 2*: All the regions of interests (ROIs) defined in atlas images in Paxinos space were firstly separated and resaved as single-ROI mask images. *Step 3*: The separated ROI mask images were transformed into individual space *via* the *InvMatrix*_*sn*_/*InvDeform*_*y*_ using 4th B-spline interpolation algorithm. The contour of each transformed ROI was identified and further refined by image erosion and expansion operator. Then, each refined ROI was given a unique integer as an index and then combined into a new atlas image. This combined atlas image was the final individual parcellations of MEMRI images.

Parcellation of rat brain images in individual space could preserve the inherent voxel intensity and ROI morphological characteristics, which would further improve the statistical sensitivity in ROI-based analyses. The automatic individual parcellation method was performed based on the stereotaxic template sets of rat brain in Paxinos space (Liang et al., 2017). The flow chart was shown in Figure 1 and detailed as follows.

Firstly, the spatial transformation from individual space to Paxinos space was calculated by registering the MEMRI image to the stereotaxic template. This registration could be performed either by affine/nonlinear transformations based on the MEMRI template image (Figure 1-*Step 1a*) (Ashburner and Friston, 1999; Zhilkin and Alexander, 2004), or by DARTEL (diffeomorphic anatomical registration through exponentiated lie) algorithm (Mak et al., 2011) based on the tissue probability maps (TPM) (Figure 1-*Step 1b*). A transformation matrix was obtained *via* template way, named *Matrix*_*sn*_, while a deformation field was obtained *via* TPM way, named *Deform*_*y*_. The inverse transformation of *Matrix*_*sn*_ or *Deform*_*y*_ was then calculated to obtain the spatial transformation from Paxinos space to individual space (Ashburner et al., 2000), and saved as *InvMatrix*_*sn*_ or *InvDeform*_*y*_.

On the other hand, the atlas images in Paxinos space were preprocessed prior to registering with an individual image (Figure 1-*Step 2*). To avoid the influences between adjacent regions, all the ROIs defined in the atlas image were separated and resaved as single-ROI mask images. In another word, each ROI was resaved as a 3D binary image, and the image intensity of voxels inside the ROI was assigned 1 and outside was 0.

Finally, transforming the atlas images into individual space to create the parcellation of individual image (Figure 1-*Step 3*). In detail, all the separated ROI mask images were registered with individual image *via* the *InvMatrix*_*sn*_/*InvDeform*_*y*_ using the 4th B-spline interpolation algorithm. Then, the ROI contour of each registered mask image was identified, and further refined by image erosion and dilation using a disc operator with one voxel radius. Next, each refined ROI was given a unique integer as an index. At last, all the reassigned ROIs were combined together to generate the final individual parcellation image.

### Animals and MRI data acquisition for evaluation

Five healthy adult Sprague–Dawley (SD) rats (male, 5; age, 10–11 week old; weight, 250–300 g) and ten modal rats with smell damage (male, 10; age, 10–11 week old; and weight, 250–300 g) were used for method evaluation.

During MRI scan, all rats were anesthetized using inhaled isoflurane/O_2_ (3% for induction and 1.5–2% for maintenance), and prostrated on a custom-made holder to minimize head motion while respiration was monitored at a rate of 50 breaths per min. For MEMRI study, all these rats were injected with manganese chloride (MnCl2·4H2O, Bio Basic Inc., Canada) dissolved in bicine [di(hydroxyethyl)glycine, Sigma-Ulrich, UK] buffer pH 7.4 in doses of 13.95 mg/kg for continuous 7 days (Tang et al., 2016).

All datasets were acquired on a 7.0T animal MRI scanner (70/16 PharmaScan, Bruker Biospin GmbH, Germany) in Nanjing, using a 38-mm birdcage rat brain quadrature resonator for radiofrequency transmission and receiving. T_2_-weighted (T2WI) data were obtained with a RARE sequence (RARE factor = 8, TR = 10493 ms, TE = 36 ms, matrix size 256^*^256^*^90, voxel size 0.14^*^0.14^*^0.3 mm, no slice gap). MEMRI data were obtained right after the last injection with a RAREVTR sequence (TR = 5500 ms, TE = 8 ms, matrix size 256^*^192^*^22, voxel size 0.14^*^0.18^*^1.2 mm^3^, no slice gap) for six repetitions. All the Bruker original images were converted to DICOM format with programs (Paravision 5.0) in the scanner. All experiments were conducted in accordance with the National Institutes of Health Guide for the Care and Use of Laboratory Animals and were approved by the Jiang Su Animal Care and Use Committee.

### Generation of individual parcellations of T2WI images

All the fifteen T2WI images were inspected and found equally of high quality in terms of the image contrast, noise level, and resolution. Firstly, all the individual T2WI images were registered into Paxinos space using DARTEL algorithm, and the corresponding *Deform*_*y*_ of each rat was obtained. Then, all the *Deform*_*y*_ were inversely transformed, and the corresponding *InvDeform*_*y*_ of each rat was obtained. On the other hand, 66 functional ROIs defined in the 3D atlas image in Paxinos space were separated and resaved into 66 binary mask images. Next, all the 66 mask images were registered with each T2WI image *via* the corresponding *InvDeform*_*y*_ repetitively using the 4th B-spline interpolation algorithm. Each registered ROI contour was identified and further refined. Finally, each refined ROI was given a unique integer as an index and combined together to generate the individual parcellations of each T2WI image.

### Generation of individual parcellations of MEMRI images

All the fifteen MEMRI images were also inspected and found equally of high quality in terms of the image contrast, noise level, and resolution. Firstly, the MEMRI image series were realigned to remove the head movement, and a mean MEMRI image was created over the six realigned volumes. Next, the mean MEMRI image was registered into Paxinos space *via* the MEMRI template image of rat brain, and the corresponding *Matrix*_*sn*_ of each rat was obtained. Then, all the *Matrix*_*sn*_ were inversely transformed, and the corresponding *InvMatrix*_*sn*_ of each rat was obtained. The preprocessing of the atlas image was the same with Section Generation of individual parcellations of T2WI images. Then, all the preprocessed ROI mask images were registered with individual mean MEMRI images *via InvMatrix*_*sn*_, and the contour was refined. Finally, each refined ROI was given a unique integer as an index and combined together to generate the individual parcellations of each MEMRI image.

### Statistical analysis of MEMRI images

For qualitative evaluation of the individual parcellations, the mean MEMRI values in dentate gyrus (*DG*) and hippocampus (*Hip*) of all the fifteen rats were extracted in individual space, respectively, and the time courses of each rat were plotted out. For qualitative comparison, all the fifteen MEMRI images were also normalized into the Paxinos space *via* the corresponding *Matrix*_*sn*_ using the 4th B-spline interpolation algorithm, and resliced to 1^*^1^*^1.5 mm^3^ (after zooming). The mean values in *DG* and *Hip* of normalized MEMRI images were also extracted and plotted out.

For quantitative evaluation of the individual parcellations, the mean values of the first MEMRI volume in *DG* and *Hip* were analyzed using SPSS software, version 19.0 (SPSS Inc. IBM, Armonk, NY, USA). The mean MEMRI values in ten modal and five healthy rats were compared by two-sample *t*-tests. Statistical significance was defined as a *p* < 0.05.

Furthermore, the accuracy of the individual parcellations was evaluated by a spatial index, named Dice similarity coefficient (*Dice*). It describes the similarity of the volume and position between the MEMRI image (*MEMRI*) and its corresponding individual parcellation image (*Parcellation*), named *Dice*_*MePa*_, as shown in Equation (1).


(1) $$\begin{array}{r} Dice_{MePa}=2\times \frac{MEMRI\cap Parcellation}{MEMRI+Parcellation} \end{array}$$

The derivation of the *Dice* is detailed previously (Gutierrez and Zaidi, 2012). The excellent agreement value of *Dice* is more than 80%. For comparison, the *Dice* was calculated between the template image and normalized MEMRI image, which was regarded as the golden standard, named *Dice*_*TempMe*_. Moreover, the previous method proposed by our group was also executed (Nie et al., 2010). In detail, each individual MEMRI image was selected as the reference, and the template image was transformed to register with it. And the *Dice* between the MEMRI image and registered template image was also calculated, named *Dice*_*MeTemp*_.

## Results

### Individual parcellations of MRI images

**Figure 2:**
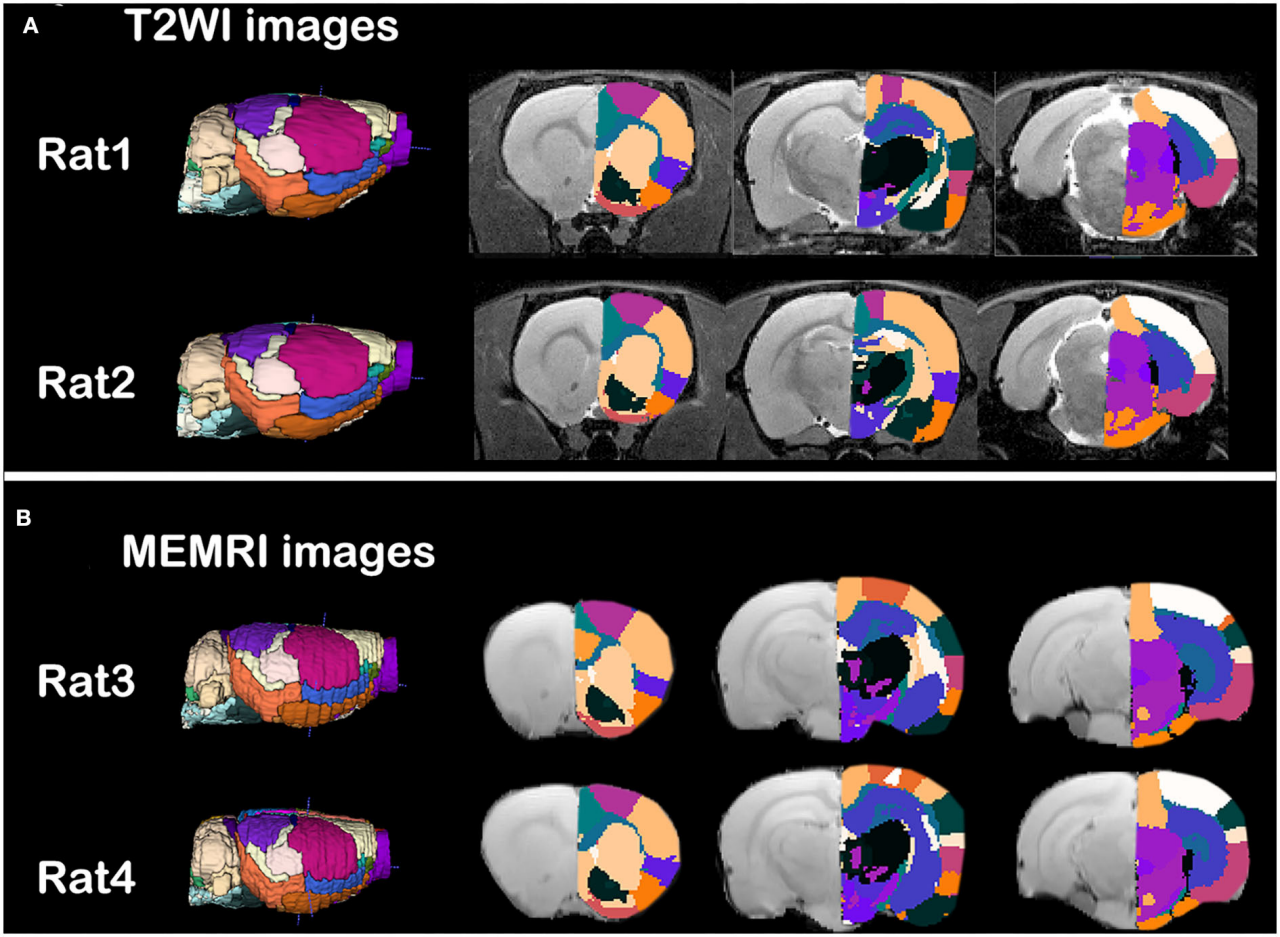
Qualitative evaluation of the individual parcellations of **(A)** T2WI images and **(B)** intercranial mean MEMRI images of rat brain. Four rats were randomly selected. Right side of the individual parcellations was shown in color scaled and superimposed on the corresponding MRI image. The T2WI/MEMRI MRI images were shown in gray scaled as background.

The individual parcellations of MRI images were shown in Figure 2. Two rats of T2WI images (Figure 2A) or MEMRI images (Figure 2B) were randomly selected. The individual parcellations were shown in color scale, while the MRI images were in gray scale. A 3D surface of individual parcellations was shown to the left, while three coronal slices were shown to the right. The individual parcellations were superimposed on the corresponding T2WI image (Figure 2A), or mean intracranial MEMRI image (Figure 2B). As illustrated in Figure 2, the parcellations were registered into the individual space accurately.

### Quantitative evaluation of individual parcellations of MEMRI images

**Figure 3:**
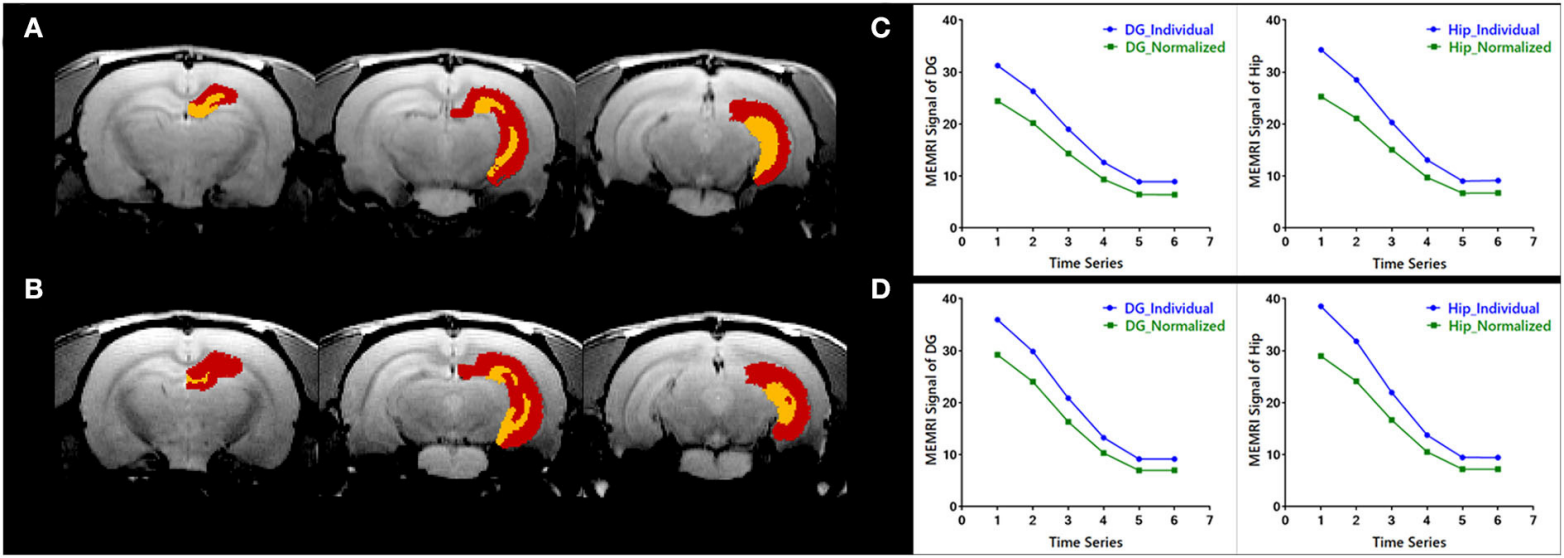
Quantitative evaluation of the individual parcellations of MEMRI images of **(A,C)** a modal rat and **(B,D)** a healthy rat. The individual parcellations were shown in color scaled in which the right dentate gyrus was yellow and the right hippocampus was red. The MEMRI images were shown in gray scaled as background. Based on the individual parcellations, the mean MEMRI signal in each ROI was extracted and the curve is shown in blue color **(C,D)**. As a comparison, the MEMRI images of these two rats were also standardized into Paxinos space, and the mean signal in each ROI was then extracted based on the atlas in Paxinos space. The signal curve extracted in traditional way was shown in green color **(C,D)**.

The evaluation of individual parcellations of two randomly selected MEMRI images was shown in Figure 3. Unilateral *DG* and *Hip* were superimposed on the first volume of MEMRI images (Figures 3A,B). The MEMRI time course in individual space was plotted out in blue color, while it in Paxinos space was in green (Figures 3C,D). As illustrated in Figure 3, although the downtrend of time courses was similar, the MEMRI voxel intensity in individual space was always higher than it in standardized space. It could be speculated that the inherent MEMRI signal could be persevered by individual parcellations.

The Dice similarity coefficients between the reference images and transformed images were listed in Table 1. The *Dice*_*TempMe*_ between the template image and normalized MEMRI image was selected as the golden standard. As illustrated in Table 1, all the *Dice* coefficients were bigger than 80%. Compared with the *Dice*_*MeTemp*_, the accuracy proposed in this study was higher.

**Table 1:** Volumetric and spatial correspondence measures.

	***Dice**_*TempMe*_* **(%)**	***Dice**_*MeTemp*_* **(%)**	***Dice**_*MePa*_* **(%)**
Whole brain	90.80 ± 0.39	89.42 ± 0.32	90.78 ± 0.20

>Dice_TempMe_ (%): Dice similarity coefficient between the template image and the normalized MEMRI image (the excellent agreement value is more than 80%).

Dice_MeTemp_ (%): Dice similarity coefficient between the individual MEMRI image and the registered template image (the excellent agreement value is more than 80%).

Dice_MePa_ (%): Dice similarity coefficient between the individual MEMRI image and the individual parcellation image (the excellent agreement value is more than 80%).

### Statistical analysis of MEMRI images

**Figure 4:**
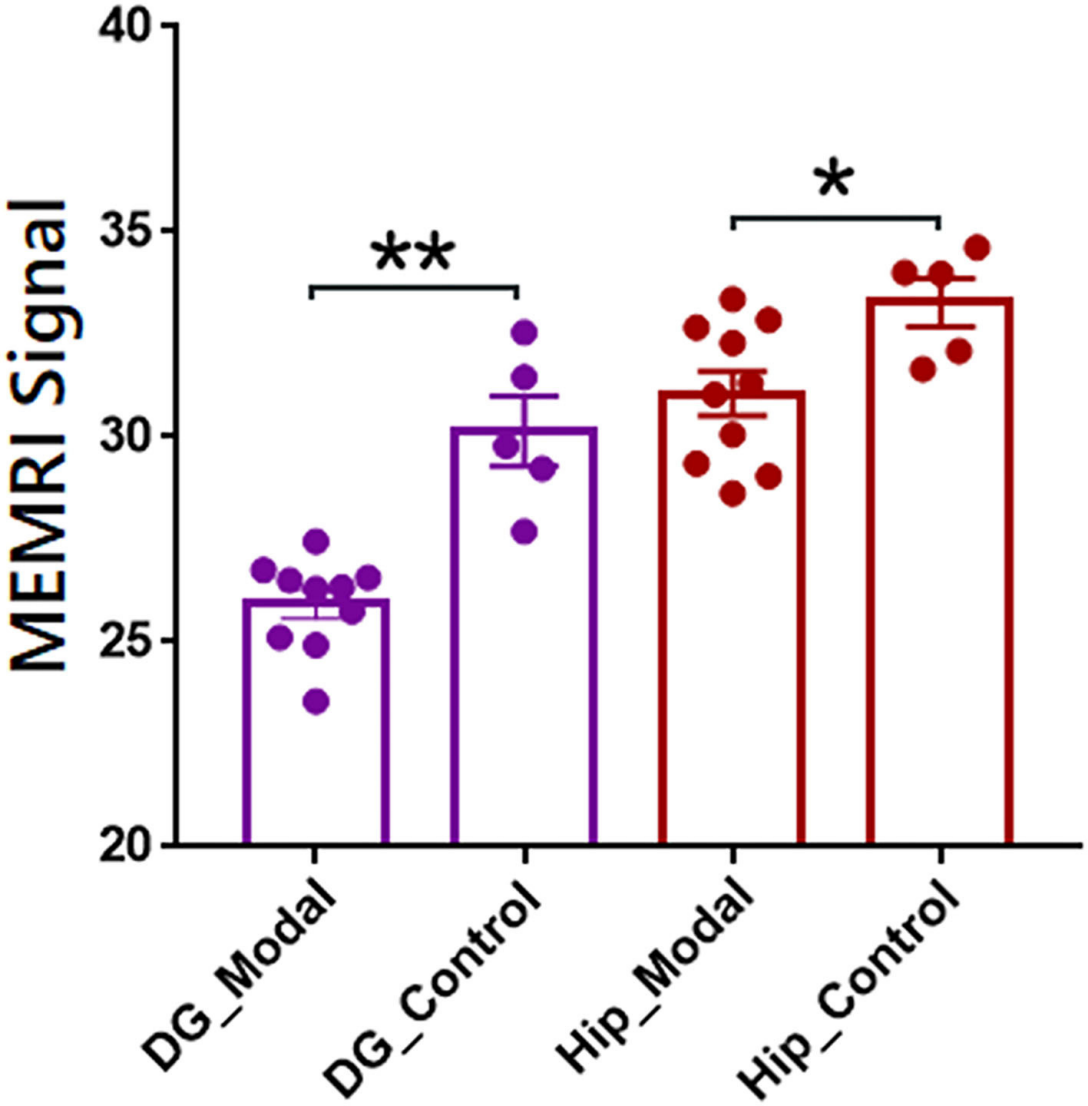
Regions of interest-based quantitative analysis results of MEMRI images between modal and healthy rats. The mean MEMRI signal in bilateral dentate gyrus (purple) and hippocampus (red) were shown as mean ± SE. **p < 0.001; *p < 0.05; DG, dentate gyrus; Hip, hippocampus.

The individual parcellations of MEMRI images were further applicate in ROI-based analyses of case-control studies. The statistical results were shown in Figure 4. The MEMRI signal of each group was presented as mean ± SD (standard deviation). The MEMRI signal in modal rats is significantly lower than the healthy rats in both *DG* (*p* < 0.001) and *Hip* (*p* < 0.05). Further studies of neural mechanisms would report in our coming article.

## Discussion

In this study, the new automatic method for individual parcellation of rat brain MEMRI images was constructed. In our proposed procedure, the individual MEMRI images were remained untransformed, so the inherent voxel intensity could be preserved. It would further be beneficial to case-control ROI-based statistical analyses.

Compared with our previous work, the individual parcellation method proposed in this study could perform more precisely. Firstly, in this study, widespread image registration methods were adopted. Especially, by DARTEL algorithm, individual images could be normalized into the template space *via* a set of tissue probability maps iteratively (Zhang et al., 2022). However, it couldn't be adopted by our previous work, for the lacking of tissue probability maps in individual space. Therefore, although the DARTEL algorithm has been recognized as a more accurate method, it couldn't be used in transforming the standard space to an individual space. On the other hand, the template has also been regarded as a representative image, so transforming the individual image to register with the template would have higher accuracy.

In spatial normalization, the MEMRI images could be firstly normalized into the standardized space *via* both template way and DARTEL way, that there was also a preferential suggestion. When the individual MRI image has a nearly isotropic resolution, the DARTEL algorithm would perform better than the template way. Otherwise, the template-based method would be more suitable, for its higher arithmetic speed. In the current study, the time course of MEMEI was preferentially considered, so the image resolution was not better enough to use the DARTEL way. Therefore, for demonstration, the MEMRI images were performed in the template way, while the corresponding T2WI structural images were in the DARTEL way.

The parcellation boundary is a problem when transforming the atlas image. Traditionally, in order to protect the ROI index, the nearest neighbor interpolation method was frequently used in atlas image transformations, so that the parcellation boundary was always dentate. To address this issue, we separated all the ROIs into a new binary mask image prior to transformation, and the 4th B-spline interpolation method was chosen. Moreover, the transformed boundary was further refined by erosion and expansion operators after transformation. By this strategy, our parcellation boundary in individual space would be smooth.

The usage and accuracy of a newly developed method are always evaluated by applications in various studies. In this study, a group of modal rats with smell damage was used, and two representative ROIs were selected. As demonstrated in the figures, compared with parcellations in standardized space, the inherent MEMRI intensity could be preserved in individual space, which was in line with the previous studies (Salehi et al., 2020; Reijonen et al., 2021; Wang et al., 2021). Image interpolation is ineluctable in image registration. However, the enhanced voxels are always subtle in MEMRI images, which would be influenced by adjacent un-enhanced voxels in image interpolation. Therefore, the image intensity in standard space is always lower than individual space. Moreover, although the downtrend of MEMRI time courses was similar, the slope is different.

Actually, the individual parcellation method proposed in this study could be applied to other imaging modalities of rat brain studies, such as functional MRI (fMRI), positron emission tomography (PET), diffusion tensor imaging (DTI), and so on. The operation was similar to MEMRI studies, except for choosing the corresponding modality template image (Figure 1-*Step 1a*). If the DARTEL algorithm was chosen (Figure 1-*Step 1b*), all the operations were the same with MEMRI studies.

Moreover, this method could also be generalized in data analyses of other species as long as there is a stereotaxic template set, such as human beings, monkeys, mice, tree shrews, and so on. In our released package, these species were all included based on our previous works of stereotaxic templates constructions.

Finally, there are some notes that should be pointed out. Firstly, the individual parcellations would be inaccurate if the corresponding image could not be successfully registered into the standardized space. Secondly, the origin of individual images should be manually set to the same point as template, such as the D3V in rat brain studies (Nie et al., 2013). Thirdly, in rodent brain studies, the image voxel size should be enlarged ten times before spatial normalization. Finally, this individual parcellation method was not suitable in cerebral tumor or infarction imaging studies. Because the sub-anatomical structures are deformed, the traditional template or TPM cannot register the image into standardized space accurately.

## Conclusions

In this study, we have constructed a new automatic method for individual parcellation of rat brain MEMRI images, which could preserve the inherent voxel intensity and further be beneficial to case-control statistical analyses. This method could also be extended to other imaging modalities, even other experiments species. It would facilitate the accuracy and significance of ROI-based imaging analyses.

## Data availability statement

The raw data supporting the conclusions of this article will be made available by the authors, without undue reservation.

## Ethics statement

The animal study was reviewed and approved by Jiang Su Animal Care and Use Committee.

## Author contributions

BN led the project. ZB, TZ, and TP created the figures and wrote the manuscript. TZ established the software. TP carried out the MEMRI data analysis. WZ and SZ made substantial contributions to the manuscript and provided critical comments. All authors contributed to the article and approved the submitted version.

## Funding

This work was financially supported by the National Natural Science Foundation of China (12175268 and 11975249).

## Conflict of interest

The authors declare that the research was conducted in the absence of any commercial or financial relationships that could be construed as a potential conflict of interest.

## Publisher's note

All claims expressed in this article are solely those of the authors and do not necessarily represent those of their affiliated organizations, or those of the publisher, the editors and the reviewers. Any product that may be evaluated in this article, or claim that may be made by its manufacturer, is not guaranteed or endorsed by the publisher.
